# Molecular Anions Move Faster than Lithium Ions as Charge Carriers in the Organic Battery Electrodes: Insights from 2,6‐Bis(diphenylamino)anthraquinone

**DOI:** 10.1002/cssc.202500785

**Published:** 2025-07-28

**Authors:** Hikaru Sano, Aya Yoshimura, Masaki Sawada, Tatsuo Noda, Yohji Misaki, Masaru Yao

**Affiliations:** ^1^ Research Institute of Electrochemical Energy National Institute of Advanced Industrial Science and Technology (AIST) 1‐8‐31 Midorigaoka Ikeda Osaka 563‐8577 Japan; ^2^ Department of Applied Chemistry Graduate School of Science and Engineering Ehime University 2‐5 Bunkyo‐cho Matsuyama Ehime 790‐8577 Japan; ^3^ Department of Technological Systems Environmental and Materials Chemistry Course Osaka Metropolitan University College of Technology 26‐12 Saiwai‐cho Neyagawa Osaka 572‐8572 Japan; ^4^ Research Unit for Materials Development for Efficient Utilization and Storage of Energy (RU:E‐USE) Ehime University 2‐5 Bunkyo‐cho Matsuyama Ehime 790‐8577 Japan; ^5^ Present address: Department of Chemistry Graduate School of Science Osaka Metropolitan University 3‐3‐138 Sumiyoshi‐ku Osaka 558‐8585 Japan

**Keywords:** battery performance, cation and anion insertion/deinsertion, lithium‐ion batteries, organic electrode materials, rate capability

## Abstract

In view of the growing demand for sustainable energy storage solutions, the potential of secondary batteries is being focused on. Lithium‐ion batteries often rely on cathode materials containing scarce rare metals. Therefore, reducing the amount of these metals in the cathode materials or developing alternatives is crucial. Minor‐metal‐free organic materials with redox activity are promising alternatives. Some organic compounds have moieties allowing the insertion and deinsertion of cations as well as anions during charging and discharging cycles. This study aims to determine if the insertion and deinsertion of monatomic cations or molecular anions offer superior rate performance in organic materials. We are focusing on 2,6‐bis(diphenylamino)anthraquinone, which, after in‐cell polymerization, accommodates the insertion and deinsertion of both cations (Li^+^) and anions (PF_6_^−^). The investigation of the reaction rates for each ion reveals that molecular anions move faster than monatomic lithium‐ions as charge carriers in the organic battery electrodes. The findings provide insights into the ion dynamics within organic electrode materials as well as shed light on anion batteries, which outperform cation batteries, such as lithium‐ion batteries.

## Graphical abstract

The suitability of molecular anions and lithium cations as carrier ions for use with organic battery materials is compared. The findings reveal that molecular anions move faster than lithium ions, suggesting that anion‐based batteries may offer superior rate‐performance. This discovery highlights the advantages of anion transport in battery technology, paving the way for more efficient and effective energy storage solutions.

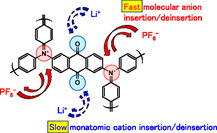

## 1 Introduction

The effective utilization of renewable energy, crucial for a sustainable society, necessitates the use of secondary batteries. Secondary batteries with high‐rate performance are essential for portable devices, computers, and vehicles such as cars and airplanes. Most such batteries contain inorganic active materials with minor metals. Organic materials are a promising alternative because they are minor‐metal free and are more abundant on earth. Therefore, the use of organic materials as active materials in batteries is highly attractive, and many researchers are active in this field.^[^ ^1^, ^2^, ^3^, ^4^, ^5^, ^6^, ^7^, ^8^, ^9^, ^10^, ^11^, ^12^, ^13^, ^14^, ^15^, ^16^, ^17^, ^18^, ^19^, ^20^, ^21^, ^22^, ^23^, ^24^ ^]^

In lithium‐ion batteries, lithium ions are typically inserted and deinserted in/from the active materials via charge/discharge reactions. However, in some lithium‐ion batteries with organic active materials, not only monatomic cations but also molecular anions,^[^ ^4^, ^5^, ^11^, ^12^, ^13^, ^14^, ^15^ ^]^ or even both, are inserted and deinserted in/form the active materials via charge/discharge reactions.

**Figure 1:**
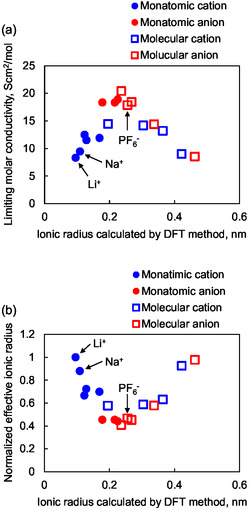
The correlation of the ionic radius, which was determined from DFT calculations with a) the limiting molar conductivity in propylene carbonate solution and b) relative value of effective ionic radius calculated from the limiting molar conductivity using Stokes's low. In this calculation, the molecular volume of each ion was first obtained from a DFT method (6‐31+G*). Then, the ion radius was estimated as the radius of the sphere having the same volume.

**Figure** **1** shows the correlation of ionic radius determined from density functional theory (DFT) calculations with limiting molar conductivity^[^ ^25^ ^]^ and the relative value of effective ionic radius calculated from the ionic radius using Stokes's law. Lithium and sodium ions are very small in their ionic radii. However, they have very low limiting molar conductivity (Figure 1a). Such a low limiting molar conductivity suggests large effective ionic radii. The difference between ionic radii calculated using DFT and effective ionic radii comes from the solvation. Monatomic ions, such as lithium and sodium ions, are solvated in the electrolyte. Figure 1 clearly shows molecular anions, such as PF_6_^−^, move faster than monatomic cations in the electrolyte.

**Figure 2:**
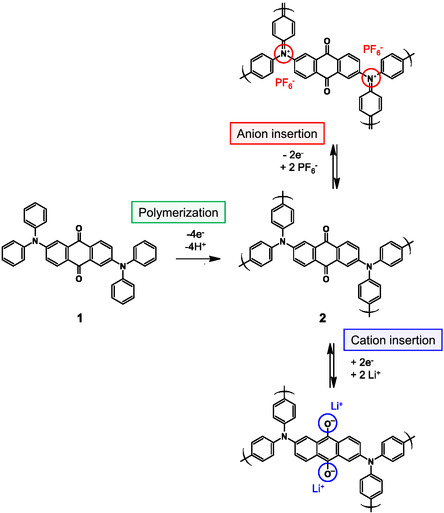
Polymerization of 2,6‐bis(diphenylamino)anthraquinone (**1**) and the charge/discharge reaction of the polymer (**2**).

Comparison of the monatomic cation and molecular anion from the viewpoint of the diffusion behavior in the electrolyte, for the case of these diffusions in the solid materials, the property of the polymer electrolyte for lithium‐ion batteries has been intensively investigated.^[^ ^26^, ^27^ ^]^ The transport number of lithium ions is around 0.3 in ordinary cases for polymer electrolyte, indicating molecular anions move faster than monatomic lithium ions in the polymer electrolyte, such as polyethylene oxide. However, to the best of our knowledge, there have been no prior studies that directly compare the diffusion behavior of cations and anions in actual electrode active materials used in batteries, probably due to the difficulty in designing a proper experimental condition. Since battery performance is significantly influenced by the electrode fabrication method, it is desirable to compare cation‐based and anion‐based materials using the same electrode. To this challenge, the use of organic active materials would be a solution to compare the diffusion characteristics between monatomic cations and molecular anions because some of the reported organic active materials can store both anions and cations as charge carriers. We focused on a single active material with both cation and anion insertion and deinsertion sites during redox reactions, specifically the polymer of 2,6‐bis(diphenylamino)anthraquinone (**1**, **Figure** **2**), which we previously confirmed,^[^ ^10^ ^]^ allows for the insertion and deinsertion of both cations and anions. In this study, we found that deinsertion occurs faster than insertion, and anions exhibit higher mobility than cations in the organic electrode material. This finding indicates that active materials using anion charge carriers are highly advantageous.

## 2 Results and Discussion

### 2.1 Initial Behavior and Cycle Performance

Prior to evaluating the rate characteristics, we evaluated and interpreted the initial charge/discharge behavior. Figure 2 illustrates the molecular structures of the initial material **1** and its polymer **2** produced by the oxidative polymerization in the cell along with the reaction formulas of the polymer **2**. The polymer **2** undergoes an anion and a cation insertions during the oxidation and reduction processes, respectively. As shown in Figure 2, the number of effective electrons during the reaction of polymerization, and anion and cation insertion/deinsertions are 4, 2, and 2. Then, the theoretical capacities are 198, 99, and 99 mAh g^−1^, respectively (**Table** **1**).

**Table 1:** Theoretical capacity under the assumption of the reaction, which is shown in Figure 2.

Reaction	Number of effective electrons	Theoretical capacity [mAh g^−1^]
Polymerization	4	198
Anion insertion/deinsertion	2	99
Cation insertion/deinsertion	2	99

**Figure 3:**
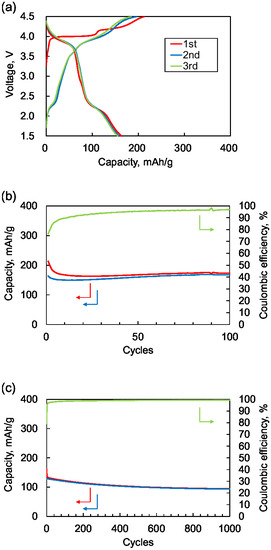
a) Charge and discharge curves for the first three cycles. Red line: 1st charge/discharge, blue line: 2nd charge/discharge, and green line: 3rd charge/discharge. Cycle performance at current density of b) 100 mA g^−1^ and c) 2000 mA g^−1^. Red: charge capacity, blue: discharge capacity, and green: Coulombic efficiency.

**Figure** **3** shows the charge and discharge curves for the initial three cycles and cycle performance. During the first charge, material **1** undergoes polymerization and anion insertion. The observed charge curve exhibits two distinct regions, one is near 4.0 V and another between 4.2–4.5 V, attributed to the polymerization and the anion insertion reaction. While the exact contribution of each region remains unclear, the capacitance at the higher potential likely corresponds to polymerization. The initial charge capacity was 210 mAh g^−1^, representing 70% of the theoretical capacity of 297 mAh g^−1^.

The subsequent discharge curve shows stepwise behaviors at around 3.9 and 2.2 V, each ≈75 mAh g^−1^. These are attributed to the anion deinsertion and cation insertion, respectively. Each is about 75% of their theoretical capacity, which assignment is further supported by similarity to previously reported voltages for anion insertion/deinsertion involving the triphenylamine structure^[^ ^8^, ^9^ ^]^ and cation insertion/deinsertion involving the quinone moieties.^[^ ^2^ ^]^

The second charge curve shows three regions at 2.2, 3.9, and 4.2–4.5 V. The region at 2.2 and 3.9 V is attributed to the cation deinsertion and anion insertion reaction, respectively, coinciding with the plateau positions of the first discharge curve. The capacity observed at 4.2–4.5 V is attributed to polymerization, suggesting the polymerization process was not fully complete during the first charge. The third charge/discharge curve closely overlaps with the second, demonstrating excellent cycling stability of the active material.

The cycle performance was remarkably stable, as shown in Figure 3b,c. For the case with 100 mA g^−1^ of current density, after 50 cycles, the discharge capacity remains ≈180 mAh g^−1^, and the Coulombic efficiency is maintained higher than 95%. For the case with 2000 mA g^−1^ of current density, the discharge capacity remains stable at 73% of the initial capacity even after 1000 cycles, and the Coulombic efficiency is over 99%.

**Figure 4:**
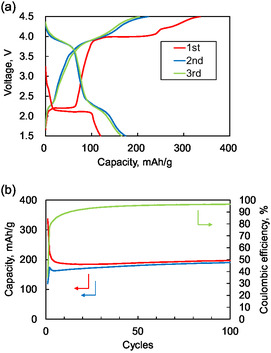
a) Charge and discharge curves for the first three cycles for the case with starts from discharging. Red line: 1st charge/discharge, blue line: 2nd charge/discharge, and green line: 3rd charge/discharge. b) Cycle performance for the case starts from discharging. Red: charge capacity, blue: discharge capacity, and green: Coulombic efficiency.

Next, we carried out the battery test, starting with discharging at the same current density (**Figure** **4**). In the initial discharge curve, only one plateau was observed at 2.2 V, which is the process of cation insertion into Material **1**, whose capacity of 100 mAh g^−1^ matches the theoretical value. In the subsequent charging, the voltage profile is divided into three regions: a plateau at 2.2 V, corresponding to cation deinsertion from Material **1**; regions at 4.0 and 4.2–4.5 V, similar to the charge‐start test with charge‐start, which is attributed to anion insertion into Material **1**; and the polymerization process of Material **1**, respectively. The obtained capacity was close to the theoretical values. For the second discharge, the profile is divided into two regions: a region at around 4.0 V with a capacity of 100 mAh g^−1^ and a region at around 2.2 V with a capacity of 100 mAh g^−1^. These regions are attributed to the reaction of anion deinsertion and cation insertion, respectively, the capacity value of which is almost the same as the theoretical capacity. The subsequent second charge exhibited two plateaus at 2.2 and 3.9–4.5 V, total capacity of around 220 mAh g^−1^, attributed to cation deinsertion, anion insertion, and polymerization. It is considered that sufficient polymerization did not proceed during the first charge, and the capacity observed during the second charge at around 4.2–4.5 V was due to polymerization. The third charge/discharge curve closely overlaps with the second, demonstrating excellent cycling stability of the active material. The charge/discharge behavior after initial discharge is very similar to that from the first charge for the case with the charging‐start condition. This is because material **1** was not polymerized at the first discharge in the case with the discharging‐start condition. The cycle performance for the case with discharge‐starting condition demonstrated excellent stability, as shown in Figure 4b. Following 50 cycles, the discharge capacity maintained a steady value of around 180 mAh g^−1^, with the Coulombic efficiency consistently exceeding 95%.

Notably, the voltage plateaus are quite flat before polymerization. However, after polymerization, the plateau of the reaction due to anion or cation insertion/deinsertion exhibits tilted voltage curves. This phenomenon has also been reported in other papers dealing with polymer active materials.^[^ ^8^, ^9^, ^10^, ^16^, ^17^ ^]^ A flat voltage profile is generally attributed to two‐phase coexistence in a single molecule. In the polymerized state, the charge and discharge curves are almost identical between the charge‐start and discharge‐start cases, indicating similar in‐cell polymerization. Although two‐stage plateaus of 50 mAh g^−1^ each are expected to be observed, they are not observed. The discharge and charge around 2.2 V after polymerization appear symmetrical, but the discharge capacity is larger than the charge capacity. The reason for this asymmetry is possibly correlated to an overpotential required for cation deinsertion after polymerization.

### 2.2 Electrochemical Impedance Spectroscopy

**Figure 5:**
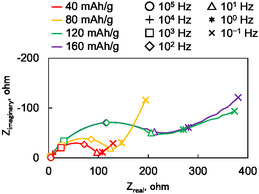
Nyquist plots of impedance spectra measured at capacities of 40, 80, 120, and 160 mAh g^−1^, over a frequency range from 10^5^ to 10^−1^ Hz.

**Figure** **5** shows Nyquist plots obtained from the impedance measurements at various capacities. Each spectrum consists of two components: a semicircle and a linear part. The impedance plots at high frequencies (10^4^–10^5^ Hz) are close to the origin and the values are very small compared to the impedance behavior at lower frequencies regions. Each vertex frequency of the semicircle is around 10^2^ Hz and each characteristic frequency of the linear part is from 10^1^ to 10^−1^ Hz, indicating they are related to ionic movements. The semicircle is in correspondence with charge‐transfer reactions, and a Warburg impedance is associated with an ion diffusion. The impedance values at high frequencies did not change drastically regardless of the depth of discharge; however, the size of the semicircle and the Warburg behavior changed between the former half (40 and 80 mAh g^−1^) and latter (120 and 160 mAh g^−1^) of the discharge process. The observation indicates that the resistance component of this cell is dominated by ionic conduction not electronic one. Based on the discharge curves shown in Figure 3a, the impedance spectra obtained at capacities of 40 and 80 mAh g^−1^ correspond to the anion insertion/deinsertion reactions, while those at 120 and 160 mAh g^−1^ correspond to the cation insertion/deinsertion reactions. The semicircle and the Warburg impedance in the anion insertion/deinsertion region are both smaller than those in the cation insertion/deinsertion region, indicating the anion‐related reaction proceeds more rapidly than the cation‐related reaction. A more comprehensive analysis will be presented in our future work.

### 2.3 Rate Performance from 1.5 to 4.5 V

**Figure 6:**
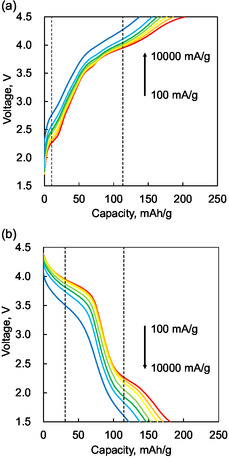
a) Charge and b) discharge rate performance. The charge and discharge voltages at the capacity indicated by the dashed lines were compared to investigate the overpotential of anion and cation insertion/deinsertion.

**Figure 7:**
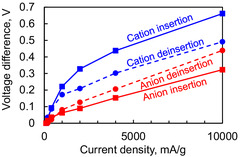
Difference in reaction voltage for anion and cation insertion/deinsertion at each current density. The difference between the charge and discharge voltages at the capacity indicated by the dashed lines in Figure 6 was calculated.

**Figure** **6** shows the charge and discharge rate performance test results, with current densities ranging from 100 to 10 000 mA g^−1^. Notably, even at 10 000 mA g^−1^ (50 C‐rate), ≈70% of the capacity at 100 mA g^−1^ (0.5 C‐rate) is retained in both charge and discharge, indicating excellent rate performance of the active material. Here, the capacity around 4 V corresponds to anion insertion/deinsertion, while that around 2.2 V to cation insertion/deinsertion. The overvoltage required for each reaction was analyzed to compare the reaction rates. For the cation deinsertion, the charge voltage at 10 mAh g^−1^ was recorded. For the anion insertion, the charge voltage at 113 mAh g^−1^ was recorded. For the anion deinsertion, the discharge voltage at 31 mAh g^−1^ was recorded. For the cation insertion, the discharge voltage at 115 mAh g^−1^ was recorded. The voltage difference at each current density relative to the values at 100 mA g^−1^ was then calculated and plotted in **Figure** **7**.

Given the excellent rate performance, we can assume negligible overvoltage at 100 mA g^−1^. Therefore, the vertical axis values in Figure 7 represent the overvoltage. Comparing the overvoltages of the cation and anion insertion and deinsertion reactions, it is evident that the anion insertion and deinsertion reactions have a smaller overvoltage. In this study, the cation is a lithium‐ion (monatomic), and the anion is PF_6_^−^ (molecular ion), which has a larger ionic radius. Consequently, the solvation/desolvation and the ion transfer process within the electrode are smoother for anion insertion and deinsertion than for cation. Our study, comparing anion and cation insertion/deinsertion within the same active material under consistent conditions, provides highly reliable results.

### 2.4 Rate Characteristics of Anion‐Related and Cation‐Related Voltage Regions

**Figure 8:**
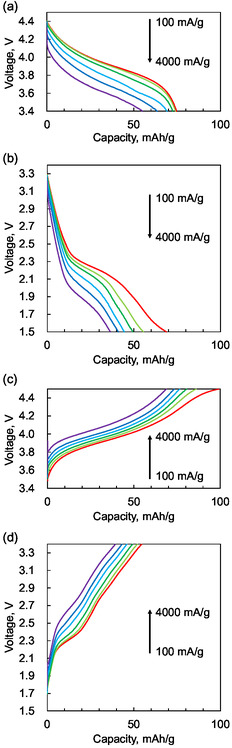
a,b) Discharge and c,d) charge rate performance. (a,c) The voltage region of 3.4–4.5 V corresponds to anion insertion/deinsertion. (b,d) The voltage region of 1.5–3.4 V corresponds to cation insertion/deinsertion.

**Figure 9:**
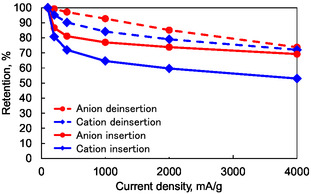
The capacity retention rates of the insertion and deinsertion reactions of anions and cations. The capacity values obtained from Figure 8 were plotted.

Next, to isolate the rate characteristics of anion and cation insertion/deinsertion, we conducted further tests within specific voltage regions: 3.4–4.5 V for anions and 1.5–3.4 V for cations. This approach aimed to avoid the coupled effects observed in the previous rate tests. **Figure** **8** shows the rate performance within these isolated voltage regions. The charge/discharge curves reveal a high‐rate capability for anion deinsertion, while cation insertion exhibits a comparatively lower rate. **Figure** **9** summarizes the capacity retention at various charge and discharge rates for each region. Notably, deinsertion reactions are faster than the insertion reactions for both anions and cations. Furthermore, anion‐related reactions are faster than the cation‐related reactions in both deinsertion and insertion. The rate performance tests divided into two voltage bands corroborate results to those in the previous chapter.

### 2.5 Cyclic Voltammetry

**Figure 10:**
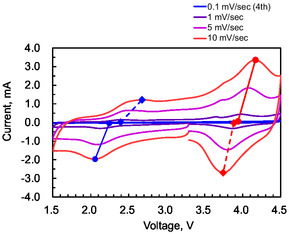
Cyclic voltammetry profiles. The scans were performed within a potential window of 1.5–4.5 V, initiated from 3.3 V in the negative direction. Four consecutive scans were conducted at 0.1 mV sec^−1^, with only the fourth scan shown. Subsequent single scans were performed at 1, 5, and 10 mV sec^−1^. Lines connect peaks corresponding to cation (2.0–2.8 V) and anion (3.6–4.2 V) insertion/deinsertion processes.

Analyzing the cyclic voltammetry behavior at varying scan rates is a powerful approach to determining reaction kinetics. For investigation of the utility of organic materials as active materials, cyclic voltammetry is sometimes employed to observe the redox behavior using low‐concentration (e.g., 10^−4^–10^−3^ mol L^−1^) solutions of the active materials. However, in this study, a two‐electrode cell with a battery electrode as the working electrode was used for cyclic voltammetry testing. That is, the experimental setup was identical to a standard charge–discharge test, which measures the capacity and voltage profile of a battery. Subsequently, **Figure** **10** shows the cyclic voltammetry results. The scan was performed four times at a scan rate of 0.1 mV sec^−1^ within a potential window of 1.5–4.5 V, followed by single scans at 1, 5, and 10 mV sec^−1^. Only the fourth scan of the 0.1 mV sec^−1^ data is presented in Figure 10. The scans were initiated from an initial potential of 3.3 V in the negative direction. The cyclic voltammetry results revealed distinct wave peaks: those around 2.0–2.8 V, assigned to cation insertion/deinsertion processes, and those around 3.6–4.2 V, assigned to anion insertion/deinsertion processes. Furthermore, connecting the peaks corresponding to the same reaction with lines, it was found that the peak position shift due to the scan rate increase is smaller for the anion‐related peaks compared to the cation‐related peaks. These observations strongly confirm faster kinetics for anion insertion/deinsertion reactions.

**Figure 11:**
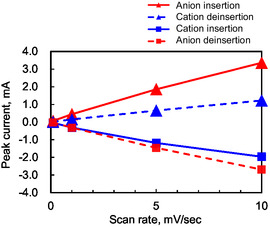
Peak current values corresponding to cation and anion insertion/deinsertion reactions at each scan rate, as derived from the cyclic voltammograms presented in Figure 10.

The reaction mechanism was elucidated by examining the correlation between scan rates and the peak current values corresponding to the anion and cation insertion/deinsertion. As shown in **Figure** **11**, a linear proportionality between the scan rate and the peak current was observed for all four redox reactions. Importantly, plotting the peak current against the square root of the scan rate revealed no linear correlation. The results strongly indicate that the redox processes are surface‐confined and adsorption‐controlled rather than diffusion‐limited. This is consistent with the conventional configuration of electrode materials deposited on the current collector.

### 2.6 Fast Anion Transport Mechanisms

As described in the introduction, anions generally exhibit weaker interactions with solvent molecules in electrolytes than cations, which contributes to the relatively small Stokes's radius and then to higher mobility of anions. In certain solid‐state systems, such as polymer electrolytes, anions also often move faster than cations reflecting the weaker interactions of anions with bulk solid‐state matrixes.

It is plausible that similar behavior occurs in organic active materials, where anions may interact weaker with the host material than cations, which would facilitate the faster diffusion of the anions. Particularly in the ion transport within organic active materials, carrier ions must overcome the electrostatic attractive interaction with polarized redox moieties. If anions have weaker electrostatic attractive interactions with these moieties, it can explain the experimental results that anions move faster than cations during charge and discharge processes, which were observed from the impedance measurements, rate performance tests, and cyclic voltammetry measurements.

## 3 Conclusion

Many organic batteries offer the significant advantage of utilizing electrodes that operate with both molecular anions and monatomic cations as carrier ions, a feat challenging to achieve with inorganic active materials. However, while it has been known that molecular anions exhibit faster movement than monatomic cations in electrolytes, detailed investigations within electrodes have been lacking. Given that electrode reactions, regardless of whether they involve monatomic cations or molecular anions, are significantly influenced by the dispersion and arrangement of battery components within the electrode, studies using electrodes with identical conditions and states are crucial. Based on our prior experience, we focused on 2,6‐bis(diphenylamino)anthraquinone. This molecule undergoes in‐cell polymerization during the initial charge, and its polymer possesses redox‐active functional groups capable of both anion and cation insertion/deinsertion. Therefore, we hypothesized that by creating an electrode using this material, we could fairly compare the insertion/deinsertion reactions of monatomic cations and molecular anions. The material polymerized in‐cell during charging, and both monatomic cations and molecular anions reacted at distinct potentials, demonstrating excellent cycling performance suitable for reaction rate comparison through rate capability evaluation. This comparison was conducted within the voltage range where both monatomic cations and molecular anions relate, as well as in separate voltage bands where each relates independently. Additionally, we obtained cyclic voltammograms of the electrode and compared the reaction rates of monatomic cations and molecular anions by analyzing the peak position shifts with varying scan rates. The results from these comparative analyses consistently showed that the reactions involving molecular anions proceeded faster than those involving monatomic cations. These results shed light on the anion batteries, which could potentially outperform cation batteries, such as lithium‐ion batteries, in terms of rate capability, offering insights for future battery development.

For future work, however, several challenges remain. 1) Given that the active material weight ratio was only 10%, some extent of ion diffusion within the electrolyte in electrodes might have influenced the observed reactions. Therefore, future studies should investigate electrodes with higher active material weight ratios. 2) While the electrical interactions between the active material moieties and career ions are crucial for ion diffusion within crystalline or amorphous active materials (our focus), sufficient room for ion diffusion is also important, which needs the optimization of molecular ion size and the room for ion diffusion in the active material. Thus, future research should assess spatial gaps within crystalline or amorphous active materials and incorporate these considerations into molecular design.

## 4 Experimental Section

4.1

4.1.1

##### Cell Construction

**Figure 12:**
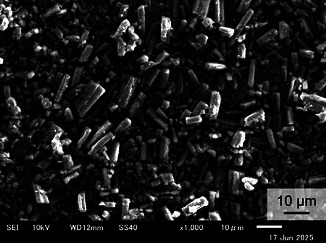
Scanning microscopic image of the powder of 2,6‐bis(diphenylamino)anthraquinone (**1**).

2,6‐Bis(diphenylamino)anthraquinone (**1,** **Figure** **12**) was purchased from Tokyo Chemical Industry (TCI, >96.0%) and ChemScene (98%). As discussed in another literature,^[^ ^8^, ^9^, ^10^ ^]^ this material was electrochemically polymerized in the cell and used as an active material. Material **1** was hand‐mixed with acetylene black (Denka as a conductive agent and polytetrafluoroethylene (PTFE, Daikin) as a binding material at a weight ratio of 1:8:1 using a mortar and pestle. The mixture was then pressed onto a stainless‐steel mesh with several megapascals of pressure to fabricate the electrodes. A two‐electrode coin‐type cell was fabricated using the prepared electrode as the working electrode, a metallic Li sheet as the counter electrode, and an LiPF_6_ solution with a concentration of 1 mol dm^−3^ (M) in a cosolvent of ethylene carbonate (EC) and dimethyl carbonate (DMC) (1:1, v/v) as the electrolyte (Mitsubishi Chemical). This fabrication procedure was conducted in a dry room with a dew point lower than −50 °C. Under the experimental conditions used in this study, the amount of electrolyte was sufficiently large (more than 0.1 cm^3^), and both anions (PF_6_^−^) and cations (Li^+^) were present in ample quantities within the cell, so any special architecture for dual‐ion battery operation was not required.

##### Electrochemical Test

For the charging and discharging test as a battery material, the conditions were set to a charge/discharge range of 1.5–4.5 V, a current density of 100 mA g^−1^, and a temperature of 30 °C. Each charge/discharge cycle began with charging unless otherwise stated. A cycling test at a high current density of 2000 mA g^−1^ was also conducted following the initial three cycles performed at a current density of 100 mA g^−1^.

In the charge rate performance test, the charge current density varied from 100 to 10 000 mA g^−1^, and the discharge current density was fixed at 100 mA g^−1^. On the contrary, in the discharge rate performance test, the discharge current density varied from 100 to 10 000 mA g^−1^, and the charge current density was fixed at 100 mA g^−1^. The rate performance test was carried out just after three conditioning cycles from 1.5–4.5 V with a charge and discharge current density of 100 mA g^−1^. A cyclic voltammetry test was also carried out using the two‐electrode coin‐type cell. The conditions were set to a scan range of 1.5–4.5 V, a scan rate of 0.1–10 mV sec^−1^, and a temperature of 30 °C.

Electrochemical impedance spectroscopy measurements were conducted under open‐circuit conditions after a 20‐min rest period at each capacity point (40, 80, 120, and 160 mAh g^−1^) during the discharge process, following several initial charge/discharge cycles with the current density of 100 mA g^−1^. The impedance measurements were performed in potentiostatic mode by applying an AC voltage with an amplitude of 5 mV over a frequency range from 10^5^ to 10^−1^ Hz at a temperature of 30 °C.

## Conflict of Interest

The authors declare no conflict of interest.

## Data Availability

The data that support the findings of this study are available from the corresponding author upon reasonable request.
